# Supplementary material for: Rhamnolipid Nano-Micelles Inhibit SARS-CoV-2 Infection and Have No Dermal or Eye Toxic Effects in Rabbits
Source: Antibiotics (Basel). 2022 Nov 4;11(11):1556. doi: 10.3390/antibiotics11111556 (PMC9686650; doi:10.3390/antibiotics11111556)
Supplement: Supplementary file 1 [file antibiotics-11-01556-s001.zip › antibiotics-1979067-supplementary.pdf]

Supplmentrary file

Table S1. Congeners composition of rhamnolipids [Rha(s)] mixture produced by *P. aeruginosa* strain LeS3 as analyzed by LC/ESI-MS in both positive and negative modes.

**Rha(s) congeners**	*m/z*							
	**Mol f**	**Mol wt**	**[M-H]$^-$**	**[M+H]$^+$**	**[M+Na]$^+$**	**[M+K]$^+$**	**[M-H+Na$_2$]$^+$**	**% abundance**
**Mono-rhamnolipid (rhamnolipid 1) congeners**								
R -C_8	$C_{14}H_{26}O_7$	306					351	12.8
R-$C_{8:1}$	$C_{14}H_{24}O_7$	304			327			25.2
R-$C_{8:2}$	$C_{14}H_{22}O_7$	302			325			29.4
R -$C_{9:1}$	$C_{15}H_{26}O_7$	318			341			0.3
R-C_{10}	$C_{16}H_{30}O_7$	334			357		379	13.6
R-$C_{10:2}$	$C_{16}H_{26}O_7$	330			353			0.12
R-C_{12}	$C_{18}H_{34}O_7$	362			385			0.24
R-$C_{12:2}$	$C_{18}H_{30}O_7$	358		359	381			2.28
R-C_{13}	$C_{19}H_{36}O_7$	376					421	0.56
R-$C_{13:2}$	$C_{19}H_{32}O_7$	372			395			0.8
R-C_{14}	$C_{20}H_{38}O_7$	390			413			0.06
R-C_{15}	$C_{21}H_{40}O_7$	404				443		0.24
R-C_8 -C_{12}, R-C_9-C_{11}, R-C_{10}-C_{10}, R-C_{12} -C_8, R-C_{11}-C_9	$C_{26}H_{48}O_9$	504	503		527	543		6.8
R-C_8-C_{14}, R-C_9-C_{13}, R-C_{10}-C_{12}, R-C_{11}-C_{11}	$C_{28}H_{52}O_9$	532	531				577	0.52
R-C_8-$C_{14:1}$, R-C_9-$C_{13:1}$, R-C_{10}-$C_{12:1}$, R-C_{11}-$C_{11:1}$, R-$C_{8:1}$-C_{14}, R-$C_{9:1}$-C_{13}, R-$C_{10:1}$-C_{12}, R-$C_{11:1}$-C_{11}	$C_{28}H_{50}O_9$	530			553			0.6
R-C_{11}-C_{16}, R-C_{12}-C_{15}, R-C_{13}-C_{14}	$C_{33}H_{62}O_9$	602		603		641		0.52
R-C_{14}-$C_{16:2}$, R-C_{15}-$C_{15:2}$, R-$C_{14:2}$-C_{16}, R-$C_{15:2}$-C_{15}	$C_{36}H_{64}O_9$	640		641				0.8
**Di-rhamnolipid (rhamnolipid 2) congeners**								
R-R-$C_{12:1}$	$C_{24}H_{42}O_{11}$	506					551	0.22
R-R-$C_{16:1}$	$C_{28}H_{50}O_{11}$	562				601		0.62
R-R-C_8-$C_{10:2}$, R-R-C_9-$C_{9:2}$, R-R-$C_{8:2}$-C_{10}, R-R-$C_{9:2}$-C_9	$C_{30}H_{50}O_{13}$	618			641	657		1.42
R-R-C_8 -C_{12}, R-R-C_9 -C_{11}, R-R-C_{10} -C_{10}, R-R-C_{12}-C_8, R-R-C_{11} -C_9,	$C_{32}H_{58}O_{13}$	650	649		673			1.6
R-R-C_{16}-$C_{16:2}$, R-R-$C_{16:2}$-C_{16}	$C_{44}H_{78}O_{13}$	815		816				0.03

Mol F, Molecular formula
Mol wt, Molecular weight
R, Rhamnose

Supplmentrary file

Table S2. Skin irritation evaluation after treatment with Rhamnolipids nano-micelles solution *

		**Control sits**								**Treated sits**							
		**Erythema and Eschar**				**Edema**				**Erythema and Eschar**				**Edema**			
		**Intact**		**Abraded**		**Intact**		**Abraded**		**Intact**		**Abraded**		**Intact**		**Abraded**	
	**Hours / Animals**	**24**	**72**	**24**	**72**	**24**	**72**	**24**	**72**	**24**	**72**	**24**	**72**	**24**	**72**	**24**	**72**
**Rhamnolipids nano-micelles (0.625 mg/ml)**	4	0	0	0	0	0	0	0	0	0	0	0	0	0	0	0	0
	5	0	0	0	0	0	0	0	0	0	0	0	0	0	0	0	0
	6	0	0	0	0	0	0	0	0	0	0	0	0	0	0	0	0

***All skin irritation ratings were 0 after 24 and 72 hours. The observations concerned erythema, eschar, and edoema. In both the undamaged and abraded areas, no symptoms or irritations were noted.**

Table S3.: **Evaluation of eye irritations following treatment with Rhamnolipids nano-micelle solution and PBS (vehicle used to disperse Rhamnolipids nano-micelles) ***

**Tested Solution**	**Tissues Examined in the eye**	**Number of rabbits**					
		4		5		6	
		RT. Untreated	LT. treated	RT. Untreated	LT. treated	RT. Untreated	LT. treated
**Rhmnolipids nano-micelles (0.625 mg/ml)**	Cornea	0	0	0	0	0	0
	Iris	0	0	0	0	0	0
	Conjunctiva	0	0	0	0	0	0

Supplmentrary file

**All eye irritation scores were 0. The observations were concerned with corneal opacity, reactivity of iris, conjunctival edema, and ocular discharge. no symptoms or irritations were noted.

Supplmentrary file

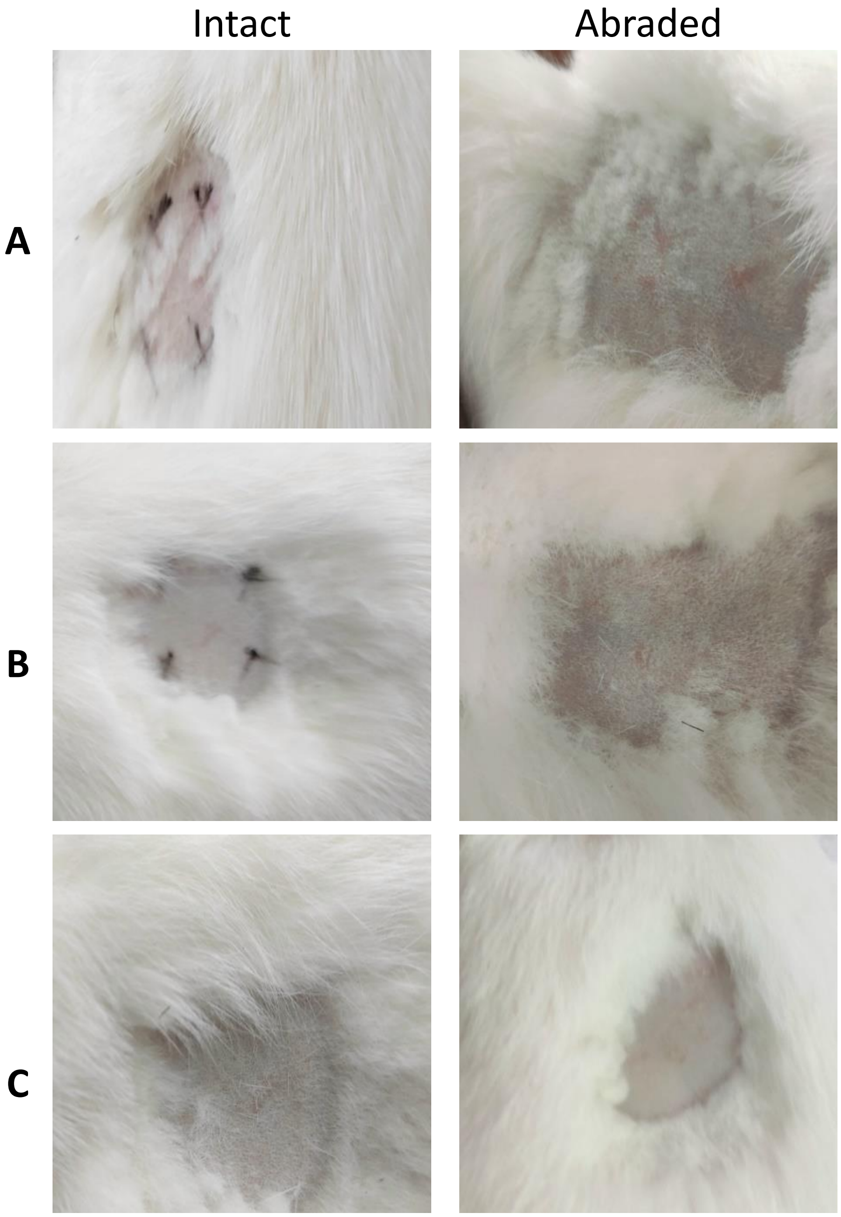

Figure S1: Representative photos of the skin irritation test for one representative rabbit for (A) Group I; untreated group, act as a control, (B) Group II; rhamnolipids nano-micelles solution (0.625 mg/ml) dispersed in PBS (10mM, pH 7.4) treated group, and (C) Group III; PBS (10mM, pH 7.4) treated group. Black lines were drawn with a non-irritating pen. No differences in score were observed after 72h between the test and control site for both abraded and intact skin.

Supplmentrary file

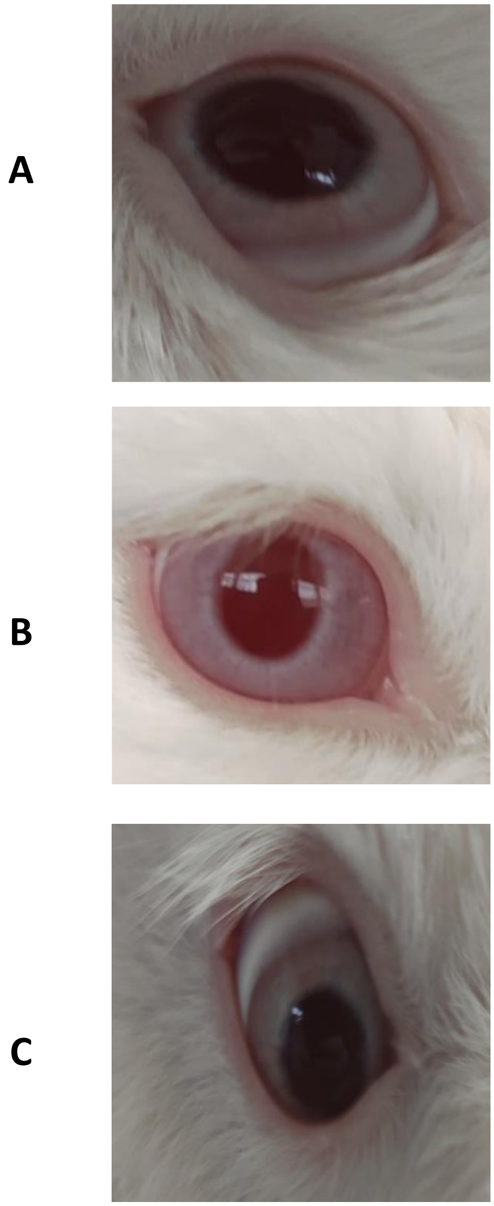

Figure S2 : Representative photos of the eye irritation test for one representative rabbit for (A) Group I; untreated group, act as a control, (B) Group II; PBS (10mM, pH 7.4) treated group, and (C) Group III; rhamnolipids nano-micelles solution (0.625 mg/ml) dispersed in PBS (10mM, pH 7.4) treated group. The cornea, iris, and conjunctiva were observed after 72 h. In this study, no differences of cornea, iris and conjunctiva were observed among different groups.

Supplmentrary file

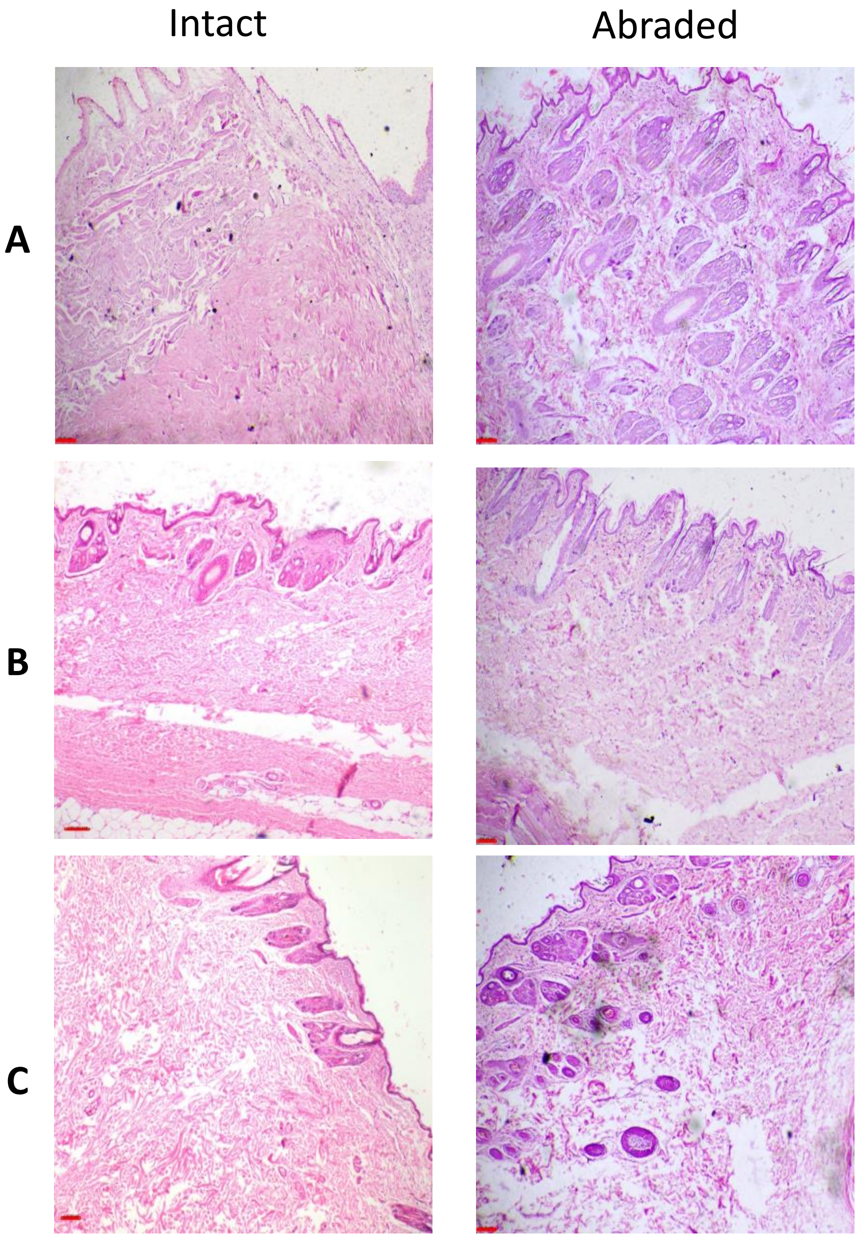

Figure S3: Representative images of H and E stained photomicrographs of skin for one representative rabbit after skin irritation test for (A) Group I; untreated group, act as a control, (B) Group II; Rhamnolipids nano-micelles solution group, and (C) Group III; PBS (10mM, pH 7.4) treated group. All groups treated with samples showed normal histological tissue identical to untreated group after 72 h of treatment with samples. All skin layers appear normal and similar to control with the absence of any sign of erosion, ulcers, necrotic cells, or inflammatory cells. Scale bar, 100 µm.

Supplmentrary file

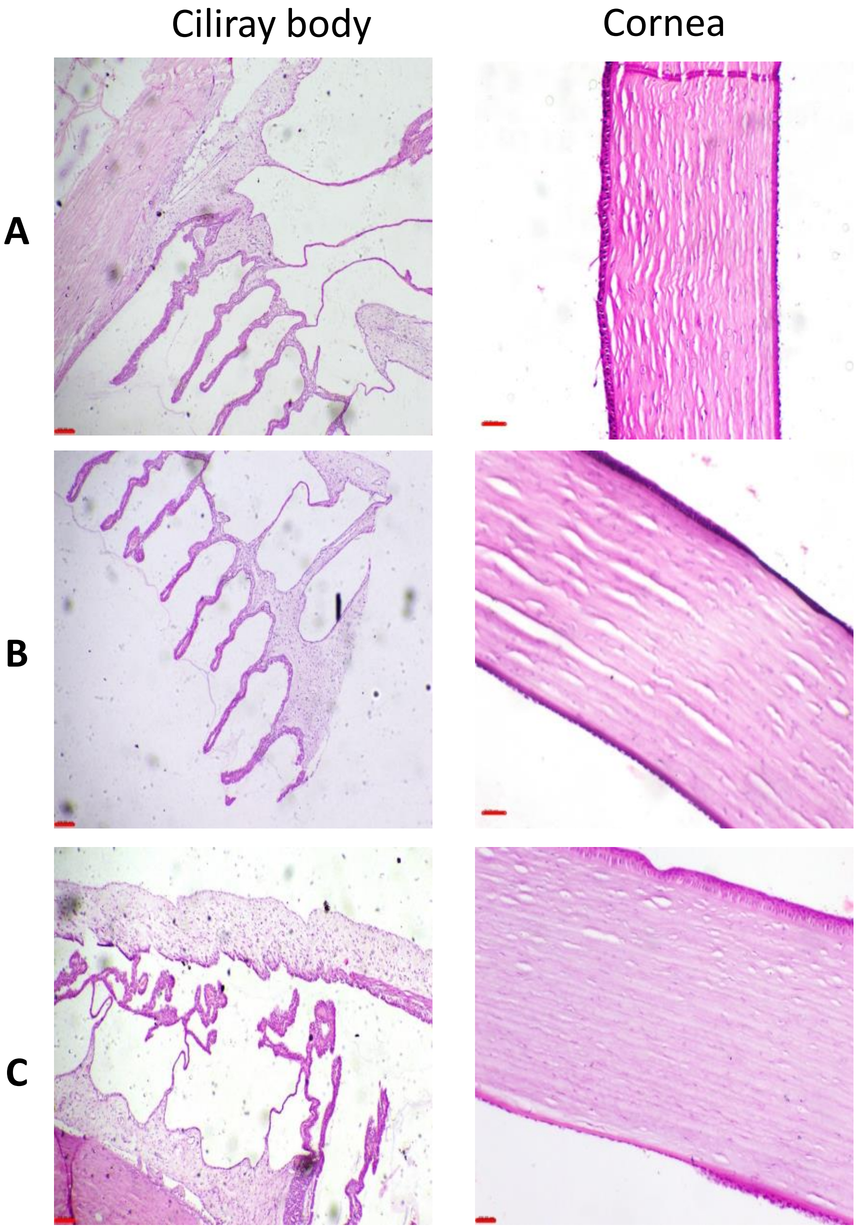

Figure S4: Representative images of H and E stained photomicrographs of eye for one representative rabbit after irritation test for (A) Group I; untreated group, act as a control, (B) Group II; rhamnolipids nano-micelles solution dispersed in PBS (10mM, pH 7.4) treated group, and (C) Group III; PBS (10mM, pH 7.4) treated group. All groups treated with samples showed normal histological tissue identical to untreated group after 72 h of treatment with samples. Cornea, fibrous connective tissue appeared normal with no signs of inflammation, erosion, ulcers or necrobiotic changes. Ciliary body appeared also normal with no cutting in the filament of ciliary body with no oedema. Scale bar, 50 μm.
